# Case Report: Acute Bulbar Palsy Plus Syndrome: A Guillain-Barré Syndrome Variant More Prone to Be a Subtype Than Overlap of Distinct Subtypes

**DOI:** 10.3389/fneur.2020.566480

**Published:** 2020-11-24

**Authors:** Qian Cao, Hong Chu, Xiujuan Fu, Jiajia Yao, Zheman Xiao, Zuneng Lu

**Affiliations:** Department of Neurology, Renmin Hospital of Wuhan University, Wuhan, China

**Keywords:** Guillain-Barré syndrome, Miller Fisher syndrome, classification, characteristics, acute bulbar palsy plus syndrome

## Abstract

**Objective:** Acute bulbar palsy plus (ABPp) syndrome is a rare regional variant of Guillain-Barré syndrome (GBS) characterized by acute bulbar palsy combined with other cranial symptoms or ataxia without limb and neck weakness. We aim to investigate characteristics of ABPp syndrome and analyze its nosological position within the GBS spectrum.

**Methods:** A patient with ABPp syndrome was reported, and previous case reports of patients who met the criteria for ABPp syndrome from the literature were reviewed.

**Results:** A total of 28 patients were included in our study. Median age was 32 years. Most of the patients (78.6%) were from Asia, and 75.0% had preceding infection. The main accompanying symptoms were ophthalmoplegia (85.7%), facial palsy (60.7%), and ataxia (50.0%). There existed asymmetric weakness in the form of unilateral facial palsy (32.1%) and ptosis (3.6%). Approximately half of the patients had albuminocytological dissociation. All the tested patients were seropositive for antiganglioside antibodies, of which the two most common were immunoglobulin G (IgG) anti-GT1a (77.3%) and anti-GQ1b (59.1%) antibodies. Over one-third of the patients who underwent electrophysiological assessment showed subclinical neuropathy beyond cranial nerves. The outcome was generally favorable as 89.3% of patients made full recovery within 5 months.

**Conclusion:** The hitherto largest case series of ABPp syndrome advances our understanding of this disease. Serologically, the presence of IgG anti-GT1a and anti-GQ1b antibodies predicts and contributes to the disease. Phenotypically, ABPp syndrome is more prone to be a separate subtype of GBS than overlap of distinct subtypes and has the potential to complement current diagnostic framework of GBS.

## Introduction

Guillain-Barré syndrome (GBS) and its principal variant, Miller Fisher syndrome (MFS), are acute immune-mediated peripheral neuropathies, which together form a continuous spectrum of both discrete and overlapping syndromes. According to a new classification system based on clinical features, GBS is divided into classic and localized forms, such as pharyngeal–cervical–brachial (PCB) weakness and bifacial weakness with paraesthesias; MFS is subclassified into incomplete forms, for example, acute ophthalmoparesis and acute ataxic neuropathy, and CNS subtypes, namely, Bickerstaff brainstem encephalitis. The majority cases of GBS could be included into this classification system (1).

The existence form of acute bulbar palsy (ABP) varies in the spectrum of GBS. It may appear with cervical–brachial weakness, when it has been referred to as PCB weakness, or occur in isolation (2), although very rarely, which has been termed incomplete PCB as a localized form of GBS at this time; additionally, ABP has also been described as a transitional symptom that either overlaps with other regional variants of GBS or evolves into generalized GBS (3). However, some GBS cases presenting with ABP in combination with other neurological symptoms, such as cranial nerve paralysis and ataxia, do not satisfy the criteria for any subtype of GBS, MFS, and their variants; polyneuritis cranialis (PNC), an oculopharyngeal subtype of GBS proposed by Wakerley et al. (4), cannot completely explain these cases either because ataxia is not allowed in PNC. In this context, Kim et al. put forward the concept of acute bulbar palsy plus (ABPp) syndrome, which is categorized to be a type of GBS manifesting with ABP plus other cranial symptoms or additional signs such as ataxia but in the absence of neck or limb weakness (5). However, the data about ABPp syndrome are still scarce due to rare clinical reports; more importantly, the problem arises as to whether ABPp syndrome can be defined on the basis of overlap between existing GBS and MFS subtypes or should be considered a separate GBS subtype.

In this article, we reported a patient with ABPp syndrome and retrospectively examined previous cases of patients who fulfilled the criteria for ABPp syndrome from the literature to further determine disease characteristics of this syndrome and analyze its nosological position within the GBS spectrum.

## Patients and Methods

We described a patient with ABPp syndrome hospitalized in the Neurology Department of Renmin Hospital of Wuhan University and searched in PubMed using various search terms related to “ABPp syndrome,” “acute bulbar palsy,” “acute oropharyngeal weakness,” and “GBS variant.” The literature with relatively sufficient details of clinical information and auxiliary examination was reviewed. All cases of patients who accorded with the diagnostic standard of ABPp syndrome from the literature were enrolled. Data were extracted and summarized for each case. The diagnostic criteria for ABPp syndrome were the following (5): (1) prominent ABP without neck and limb weakness; (2) manifestation of other cranial involvements or gait ataxia or both; (3) compliance with the general features of GBS (acute monophasic illness pattern, <4 weeks of reaching the clinical nadir, no accompaniment of upper motor neuron signs, etc.); and (4) absence of identified alternative diagnosis, including, but not limited to, cerebral vascular disease, brain tumor, myasthenia gravis, inflammatory myopathy, and botulism.

## Case Presentation

A previously healthy 52-year-old woman presented to our hospital with a sudden diplopia after waking up in the morning 3 days ago, especially when looking forward or left. Her voice was low pitched and hoarse. She could not swallow properly, and fluids regurgitated through her nose when she drank water. She also complained of dizziness and unsteadiness. She did not have limb weakness and bowel or bladder difficulties. She had symptoms, including nasal congestion, sore throat, and fever to 38°C, suggestive of an upper respiratory tract infection (URTI) 6 days before the neurological symptoms. On admission, she was afebrile and mentally alert. She had partially abducens nerve palsies bilaterally and severe dysphagia to both liquids and solids. There was bilateral paralysis of the soft palate and loss of pharyngeal reflex. Her pupils were symmetric and of a normal size, reacting briskly to light. Corneal reflexes were intact bilaterally, and examination of the facial nerves was normal. There was no weakness of neck or limb muscles. All the tendon reflexes were depressed, and the plantar responses were flexor. Sensory examination was normal, and Romberg test was positive. There were no cerebellar signs or evidence indicative of autonomic or sphincter dysfunction.

Laboratory studies, including complete blood count, routine biochemical tests, hemoglobin A1c, thyroid function, tumor markers, serum antiacetylcholine receptor antibody, antineutrophil cytoplasmic antibodies, and antiextractable nuclear antigen antibodies were unremarkable. Diffusion-weighted imaging showed no evidence of acute infarctions. A cerebrospinal fluid (CSF) analysis performed on day 3 of the illness revealed no white blood cells, a mildly elevated total protein concentration (49 mg/dl), and a normal glucose concentration (3.46 mmol/L). A screening test for antiganglioside antibodies by Kindstar company showed immunoglobulin G (IgG) anti-GQ1b and anti-GT1a antibodies positive. Nerve conduction study (NCS) and repetitive nerve stimulation performed on day 3 of the illness were normal. Serial NCS on day 11 demonstrated decreased persistence of F-wave of left ulnar and bilateral median nerves, as well as reduced amplitude of sensory nerve action potential (SNAP) in the limbs. The results of motor conduction study were otherwise normal (Table 1).

**Table 1:** Motor and sensory nerve conduction studies of case 1.

	**Day 3**		**Day 11**	
	**R**	**L**	**R**	**L**
**Motor conduction studies**				
Median nerve				
DML (ms)	3.2	2.8	3.2	2.8
CMAP (mV)	7.9	7.7	7.4	6.8
CV (elbow-wrist, m/s)	57	61	58	57
F-wave latency	25.6	25.5	22.6	24.5
F-wave persistence	90%	80%	**62.5%**	**50%**
Ulnar nerve				
DML (ms)	2.4	2.2	2.2	2.6
CMAP (mV)	7.9	8.6	8.1	7.6
CV (below elbow-wrist, m/s)	65	65	54	61
F-wave latency	25.6	24.4	24.7	23.5
F-wave persistence	100%	95%	100%	**68.8%**
Tibial nerve				
DML (ms)	3.3	3.3	3.3	4.1
CMAP (mV)	11.3	9.0	11.3	12.3
F-wave latency	47.3	45.6	49.0	47.8
F-wave persistence	100%	100%	100%	100%
Peroneal nerve				
DML (ms)	3.5	3.4	2.9	3.3
CMAP (mV)	4.1	4.3	4.4	5.9
CV (fibular neck-ankle, m/s)	46	49	45	46
**Sensory conduction studies**				
Median nerve				
Peak latency (ms)	1.9	1.9	2.2	2.1
SNAP (μV)	23.2	28.0	**16.8**	**20.6**
CV (wrist-index finger, m/s)	60	61	58	60
Ulnar nerve				
Peak latency (ms)	1.6	1.6	1.8	1.9
SNAP (μV)	21.1	22.7	**12.8**	**15.1**
CV (wrist-little finger, m/s)	60	59	60	57
Radial nerve				
Peak latency (ms)	ND	ND	2.1	2.3
SNAP (μV)	ND	ND	**14.5**	17.6
CV (forearm-snuff box, m/s)	ND	ND	50	45
Sural nerve				
Peak latency (ms)	2.3	2.0	2.3	2.1
SNAP (μV)	16.5	15.8	11.4	13.0
CV (calf-ankle, m/s)	51	54	52	52
Superficial peroneal nerve				
Peak latency (ms)	1.7	1.8	1.7	1.7
SNAP (μV)	26.5	29.9	**16.7**	**14.8**
CV (lower leg-ankle, m/s)	57	54	52	53

*DML, distal motor latency; CMAP, compound muscle action potential; CV, conduction velocity; SNAP, sensory nerve action potential; ND, not done*.

*Abnormal values are highlighted in bold*.

The patient received intravenous immunoglobulin (IVIg) treatment for 5 days (0.4 g/kg/day) on day 5 of the illness. A nasogastric tube was also inserted because of severe dysphagia. She responded well to IVIg. Her bulbar palsy, diplopia, and ataxia started to improve 4 days later and almost completely recovered at discharge on day 16 of the illness except mild blurred vision. She had no special complaints at telephone follow-up 1 month later and declined the invitation to hospital for another NCS, making further assessment of previously abnormal SNAP impossible.

## Results

With the additional 27 patients identified in 13 English-language articles (3, 5–16), a total of 28 cases were included in our descriptive analysis (Table 2).

**Table 2:** Demographic, clinical, laboratory, and electrophysiological characteristics of 28 patients.

**Case**	**Age/ sex**	**Areas of residence**	**Antecedent infection**	**Initial symptoms**	**Symptoms and signs other than ABP**	**CSF: cell count (μl)/protein (mg/dl) (time)**	**Antiganglioside antibodies**	**EDX (abnormal parameters) (time)**	**Specific treatment**	**Outcome (time after onset, weak)**
1	52/F	Asia	URTI	Diplopia, dysarthria, dysphagia	External OP, hyporeflexia, sensory abnormality, gait ataxia	0/49 (day 3)	IgG anti-GT1a, GQ1b	NL (day 3), Abn (SNAP) (day 11)	IVIg	FR (4.0)
2 (6)	22/M	Asia	GI	Nasal speech	Internal OP, areflexia	NL (time unknown)	IgG anti-GQ1b	NL (time unknown)	None	FR (several weeks, exact time unknown)
3 (7)	37/M	Europe	GI	Gait ataxia, numbness of limbs	Hyporeflexia (upper limbs)/areflexia (lower limbs), sensory abnormality, gait ataxia	NL (day 5)	IgM anti-GD3	Abn (DML, SCV) (day 5), NL (1 month later)	IVIg	FR (1.4)
4 (5)	52/F	Asia	URTI	Gait ataxia	Areflexia, sensory abnormality, gait ataxia	NL (day 3)	IgG anti-GT1a, GQ1b	NL (day 3)	IVIg	FR (2.0)
5 (5)	20/F	Asia	GI	Gait ataxia	External OP, unilateral FP, areflexia, gait ataxia	NL (day 2)	IgG anti-GT1a, GQ1b	Abn (Blink reflex) (day 2)	IVIg	FR (6.0)
6 (5)	31/F	Asia	URTI	Dysarthria	Areflexia, gait ataxia	NL (day 6)	IgG anti-GT1a, GQ1b	NL (day 6)	IVIg	FR (3.0)
7 (5)	54/F	Asia	None	Dysarthria	External OP, unilateral FP, areflexia, sensory abnormality, gait ataxia	NL (day 2)	IgG anti-GT1a, GQ1b, IgM anti-GT1a, GQ1b	NL (day 3)	IVIg	FR (7.0)
8 (5)	26/F	Asia	GI	Diplopia	External OP, unilateral FP, areflexia, sensory abnormality, gait ataxia	NL (day 2)	IgG anti-GT1a, IgM anti-GT1a	NL (day 3)	IVIg	FR (16.0)
9 (5)	21/M	Asia	None	Dysarthria, dysphagia	External OP, unilateral FP, areflexia,	ND	IgG anti-GT1a, GQ1b	NL (day 2)	IVIg	FR (7.0)
10 (5)	27/M	Asia	GI	Diplopia	External/internal OP, unilateral FP, areflexia, sensory abnormality, gait ataxia,	+(day 1)	IgG anti-GT1a	Abn (Blink reflex) (day 9)	IVIg	Not fully recovered after 30 weeks
11 (5)	65/F	Asia	None	Dysarthria	External OP, areflexia, sensory abnormality, gait ataxia	+ (day 3)	IgG anti-GT1a	Abn (DML, SNAP, SCV) (day 4)	IVIg	FR (6.0)
12 (5)	18/M	Asia	GI	Diplopia, nasal speech	External OP	NL (day 7)	IgG anti-GT1a	NL (day 18)	None	FR (6.0)
13 (5)	38/M	Asia	URTI	Dysarthria	External OP, areflexia, sensory abnormality, gait ataxia	NL (day 2)	IgG anti-GD1a, GT1a	Abn (SNAP) (day 10)	IVIg	FR (8.0)
14 (5)	20/M	Asia	GI	Numbness of limbs	External OP, unilateral FP, areflexia, sensory abnormality, gait ataxia	NL (day 2)	IgG anti-GQ1b, GT1a, GM2	NL (day 4)	IVIg	FR (4.0)
15 (8)	13/F	Asia	URTI	Dysphagia, nasal speech, unilateral FP	Unilateral FP, areflexia	ND	ND	Abn (CAMP, MCV) (time unknown)	None	FR (6.0)
16 (9)	20/M	Asia	None	Diplopia	External/internal OP	NL (day 6), –/96 (day 14)	ND	NL (day 8)	IVIg	FR (20.0)
17 (10)	52/M	Europe	URTI	Diplopia	External OP, FP	–/78 (time unknown)	IgG anti-GQ1b	Abn (MCV) (time unknown)	IVIg	FR (8.0)
18 (11)	10/M	Europe	Fever	Left ptosis, diplopia, dysarthria	External/internal OP, FP, masticatory muscle and tongue weakness	NL (time unknown)	ND	Abn (DML, facial nerves) (time unknown)	Steroids, IVIg	FR (8.0)
19 (13)	67/F	Asia	URTI	Diplopia, numbness of limbs	External OP, unilateral FP, sensory abnormality	2/70 (day 10)	ND	Abn (SNAP) (day 13)	None	FR (3.0)
20 (13)	33/M	Asia	URTI	Diplopia, FP, dysarthria	External OP, FP	0/118.5 (day 9)	ND	Abn (DML) (day 12)	IVIg	FR (6.0)
21 (13)	47/M	Asia	URTI	Unilateral FP	External OP, FP, sensory abnormality	0/15.7 (day 7)	IgG anti-GQ1b	NL (time unknown)	None	completely recovered within 1 year
22 (12)	54/M	Europe	URTI	Dysarthria, dysphagia	FP, masticatory muscle and tongue weakness	0/54 (time unknown)	IgG anti-GM3, GD1a, GT1b	Abn (Blink reflex) (time unknown)	IVIg	Mild improvement after 1 year
23 (14)	23/F	Asia	URTI, GI	Numbness of limbs	Unilateral FP, sensory abnormality, gait ataxia	3/217 (day 5)	IgG anti-GQ1b, GT1a	Abn (SNAP, SCV) (day 6)	PE	FR (exact time unknown)
24 (3)	34/F	Asia	URTI, GI	Dysarthria, dysphagia, numbness of limbs	External OP, FP, areflexia, sensory abnormality	ND	IgG anti-GM1b, GT1a, IgM anti-GM1b, GT1a	ND	PE	Mild OP (5 months)
25 (3)	19/M	Asia	None	Dysarthria, dysphagia, diplopia	External OP, areflexia	ND	IgG anti-GT1a	ND	PE	Mild OP (3.0)
26 (3)	29/F	Asia	None	Dysarthria, dysphagia, numbness of limbs	External OP, FP, areflexia, sensory abnormality	ND	IgG anti-GT1a, GQ1b, GM1b, IgM anti-GM1b, GT1a, GQ1b, GalNAc-GD1a	ND	None	Mild OP (4.0)
27 (15)	49/M	Europe	GI	Dysarthria, dysphagia, numbness of limbs	Areflexia, sensory abnormality, gait ataxia	+ (time unknown)	IgG anti-GT1a, GQ1b	ND	None	FR (exact time unknown)
28 (16)	41/F	Europe	Post-partum	Dysarthria, dysphagia, gait ataxia	External OP, FP, areflexia, gait ataxia	+ (time unknown)	ND	NL (time unknown)	None	FR (2.0)

*URTI, upper respiratory tract infection; GI, gastrointestinal illness; OP, ophthalmoplegia; FP, facial palsy; CSF, cerebrospinal fluid; EDX, electrodiagnostic study; DML, distal motor latency; CMAP, compound muscle action potential; MCV, motor conduction velocity; SNAP, sensory nerve action potential; SCV, sensory conduction velocity; ND, not done; NL, normal; Abn, abnormal; IVIg, intravenous immunoglobulin; PE, plasma exchange; FR, full recovery*.

*Data unavailable are labeled “–;” CSF characteristic of albuminocytological dissociation without specific values is labeled “+;” Case 1 is reported in this article*.

### Demographic and Clinical Characteristics

The median age was 32 years (range, 10–67 years) for 15 (53.6%) men and 13 (46.4%) women. ABPp syndrome was regional, predominating in Asia (22, 78.6%). Of the 28 patients, URTI occurred before symptom onset in 10 (35.7%) patients, gastrointestinal illness (GI) in 8 (28.6%), both infections in 2 (7.1%), fever of unknown origin in 1 (3.6%), postpartum status in 1 (3.6%), and no antecedent illness in 6 (21.3%).

Despite bulbar paralysis being the most prominent feature, initial symptoms varied in different cases. The most frequent was dysarthria (15, 53.6%), followed by diplopia (10, 35.7%), dysphagia (9, 32.1%), and numbness of limbs (7, 25.0%). Other symptoms at onset included gait ataxia (4, 14.3%), nasal speech (3, 10.7%), facial palsy (3, 10.7%), and ptosis (1, 3.6%). Besides ABP, the concomitant neurological findings during the disease course were external ophthalmoplegia (20, 71.4%), areflexia/hyporeflexia (18, 64.3%), facial palsy (17, 60.7%), gait ataxia (14, 50.0%), sensory abnormality (14, 50.0%), internal ophthalmoplegia (4, 14.3%), and masticatory muscle and tongue weakness (2, 7.1%). Of the 17 patients with facial palsy, nine patients were unilateral and the remaining eight bilateral. Of the 20 patients with external ophthalmoplegia, one patient had unilateral ptosis.

### Laboratory and Electrophysiological Examination

CSF was analyzed in 23 patients, and albuminocytological dissociation was found in 11 (47.8%) patients. Twenty-two patients underwent serological assay, and antiganglioside antibodies were identified in all the patients tested for. IgG anti-GT1a antibody (17, 77.3%) was the most frequent, followed by IgG anti-GQ1b (13, 59.1%).

Electrodiagnostic studies were conducted in 24 patients and found to be abnormal in 13 (54.2%) patients. Four (16.7%) patients had facial nerves involvement in terms of abnormal blink reflex and prolonged distal motor latency (DML). The remaining nine (37.5%) patients had varying degrees of abnormalities in limb NCS. Four (16.7%) patients showed reduced SNAP amplitude or sensory conduction velocity (SCV), indicative of impairment of sensory nerves. Two (8.3%) patients showed sensorimotor polyneuropathy with prolonged DML and decreased SNAP amplitude or SCV. Three (12.5%) patients displayed motor nerve conduction abnormalities, including prolonged DML and reduced compound muscle action potential (CMAP) amplitude or motor conduction velocity (MCV). In the case series, specific data of NCS were available only in four patients (case 1, 2, 3, and 20), one of which was normal and the other three abnormal. The three subjects (case 1, 3, and 20) with abnormal limb NCS were classified as equivocal by the latest electrodiagnostic criteria set for GBS proposed by Uncini and colleagues (17).

### Treatment and Outcome

More than half of the patients (16, 57.1%) received IVIg treatment, whereas three (10.7%) patients underwent plasma exchange and one (3.6%) patient was treated with IVIg combined with steroids. The remaining eight patients were treated conservatively.

The majority of patients (25, 89.3%) had a good prognosis with almost complete recovery within 5 months after onset. Neurological deficits persisted in two patients for more than 7 months, and one of them eventually recovered within 1 year. Only in one patient there was no significant improvement even after 1 year. Whether the immunotherapy was adopted had a marginal impact on the prognosis in that the proportion of patients who recovered within 5 months of onset in immunotherapy group (18, 90.0%) was similar to that in the conservative care group (7, 87.5%).

## Discussion

There were many similarities in clinical features and auxiliary examinations among patients in this case series, although individual in the pattern of neuropathy. A striking commonality was an acute onset of bulbar dysfunction in association with ocular weakness, facial palsy, or ataxia. Cranial nerve paralysis was usually bilateral, although unilateral facial palsy was noted in nine patients and unilateral ptosis in one. The disease course was monophasic, and three quarters of patients had preceding infection, the majority of which involved respiratory or gastrointestinal system. CSF albuminocytological dissociation observed in 47.8% of the subjects was partially in line with a previous study, which demonstrated that elevated CSF protein without pleocytosis was present in nearly half of GBS patients on the 1st day after onset and ~90% after 14 days (18). The prognosis was generally good regardless of whether immunotherapy was adopted. Based on these evidences, the above-mentioned 28 patients can be considered a variant of GBS, similar to—yet distinct from—classic MFS.

Our study revealed that facial palsy, constituting nearly two-thirds of the subjects, was relatively more common in ABPp syndrome than previously reported (5), and half of the patients with facial palsy were bilateral. In general, facial weakness occurs in ~50% of patients with GBS during disease course and is frequently bilateral (19). Bifacial weakness with paresthesias (BFP), an unusual subtype of GBS, is characterized by facial diplegia frequently associated with distal limb paresthesias but in the absence of limb weakness (20). The eight patients with bifacial weakness in our series, however, could not be diagnosed as BFP, although bulbar symptoms could be occasionally found in a minority of BFP patients (21). All these patients but one had ocular symptoms, and two of them also had masticatory muscle and tongue weakness, whereas such symptoms have not been present in the hitherto largest case series of patients with BFP (21). In addition, BFP is not associated with the generation of antiganglioside antibodies (20), yet antiganglioside antibodies were demonstrated in all the patients with bifacial weakness who were tested for in our series.

The current study also found that IgG anti-GT1a and anti-GQ1b antibodies were the two most common antibodies in the disease. The pattern of neuropathy in ABPp syndrome can be explained partially by the anatomical distribution of gangliosides. The GQ1b gangliosides are highly expressed in oculomotor, trochlear, and abducens nerves as well as muscle spindles in the limbs and thus could account for ophthalmoplegia and ataxia in patients with ABPp syndrome (22); similarly, prominent bulbar weakness in ABPp syndrome could be attributed to GT1a gangliosides that are abundant in glossopharyngeal and vagal nerves (23). Nonetheless, the positive rate of anti-GT1a antibodies in our study was not identified 100% as reported in a previous study (5). A likely reason for this is that the antiganglioside screening panel in some negative cases is not wide enough to include anti-GT1a antibodies. Furthermore, anti-GT1a antibodies are not absolutely associated with bulbar weakness in that anti-GM1b, anti-GQ1b, and anti-GM2 antibodies have also been found in GBS patients with bulbar palsy (15, 24, 25); only half of the patients with PCB weakness carry anti-GT1a antibodies, and a quarter display antibodies against GM1 or GD1a (26). Finally, anti-GQ1b antibodies are recognized to cross-react with GT1a, which might explain why some patients with anti-GQ1b antibodies, such as MFS, often develop bulbar weakness (27, 28).

Our study indicated subclinical neuropathy beyond cranial nerves because limb NCS was abnormal in over one-third of the tested patients, despite restricted weakness to territories served by motor cranial nerves. These results corroborate those of Kim et al. who found that two of 11 patients with ABPp syndrome had abnormal NCS findings (5); furthermore, the presence of subclinical limb neuropathy supports the current view that GBS forms a continuous spectrum of related disorders, in which the pattern of weakness and distribution of peripheral nerve involvement vary between patients. In this series, there was considerable variability in the timing of electrophysiological testing among patients, and none of the patients but two (case 1 and 3) performed serial assessment. Besides, specific data of NCS were not provided in most cases in the literature. Therefore, it was not possible to make an accurate electrodiagnosis of GBS subtypes (29–31). However, the presence of antiganglioside antibodies in all the tested patients prefers axonal form of neuropathy (32, 33), although more detailed serial electrodiagnostic studies are required.

Our study demonstrated that ophthalmoplegia, facial palsy, and ataxia were the most common accompanying symptoms of ABPp syndrome. Accordingly, we attempted to categorize all the patients on the basis of different combinations of core symptoms (Table 3). Overall, the vast majority of patients displayed bulbar weakness plus ophthalmoplegia and/or ataxia, which were often associated with facial palsy (unilateral or bilateral). It seems that ABPp syndrome might represent an overlap between incomplete form of PCB weakness (acute pharyngeal weakness) and MFS or its less extensive subtypes (acute ophthalmoparesis and acute ataxic neuropathy). This conjecture is serologically supported by the presence of IgG anti-GT1a and anti-GQ1b antibodies in more than half of the cases.

**Table 3:** Comparison of two classification methods for enrolled patients.

**Classification in this study**	**Classic classification**	**Case number**	**Number of cases (%)**	**Notes**
ABP plus OP(with or without FP)	Acute pharyngeal weakness overlapping acute ophthalmoparesis	2, 9, 12, 16, 17, 19, 20, 21, 24, 25, 26,	11 (39.3%)	7 cases of FP (2 of them are unilateral)
ABP plus OP and ataxia(with or without FP)	Acute pharyngeal weakness overlapping MFS	1, 5, 7, 8, 10, 11, 13, 14, 28	9 (32.1%)	6 cases of FP (5 of them are unilateral)
ABP plus ataxia(with or without FP)	Acute pharyngeal weakness overlapping acute ataxic neuropathy	3, 4, 6, 23, 27	5 (17.9%)	1 case of FP (unilateral)
ABP plus FP; ABP plus FP, masticatory muscle and tongue weakness; ABP plus OP, FP, masticatory muscle and tongue weakness	Unclassifiable	15, 18, 22	3 (10.7%)	3 cases of FP (1 of them is unilateral)

*ABP, acute bulbar palsy; OP, ophthalmoplegia; FP, facial palsy; MFS, Miller Fisher syndrome*.

However, it is not the case from phenotypic point of view for three reasons. First, it has been found by a prospective study that MFS accounted for 5% of GBS cases and PCB only 3% (34); therefore, the frequency of incomplete forms of MFS and PCB is reasonably even lower although not reported in detail. Also noteworthy is that more than half of patients in our study had facial palsy, but this symptom has not as yet been reported to coexist with acute pharyngeal weakness (2, 15, 24) and only rarely been reported to associate with acute ophthalmoparesis and acute ataxia neuropathy (35, 36). Considering the improbability of an overlap between two rare subtypes that are not usually connected with facial palsy, ABPp syndrome cannot be simply explained by overlap mechanism. Second, in the acute pharyngeal weakness overlapping MFS group, six of nine patients developed facial palsy and five of them are unilateral; asymmetric cranial neuropathy, although permitted and well-recognized, is uncommon in GBS (37, 38). Moreover, in two large case series of patients with MFS (27, 28), 22–32% had facial palsy and 17–26% had bulbar palsy, but it was difficult to know with certainty whether and how often the two symptoms could occur simultaneously in MFS. Given the small probability of coexistence of bulbar and facial palsy in MFS as well as asymmetric facial palsy, the pattern of neuropathy in ABPp syndrome goes beyond what would be commonly predicted from the overlap between acute pharyngeal weakness and MFS. Finally, three patients cannot be classified into GBS and MFS subtypes either in isolated or overlapping form. Consequently, instead of viewing it as overlap of distinct subtypes, it is more plausible to consider ABPp syndrome as a separate subtype of GBS, which is intermediate between GBS and MFS.

The relationship between ABPp syndrome and PNC warrants brief discussion. PNC has been used to describe diseases that affect several cranial nerves regardless of etiology. Some cases have disease characteristics and laboratory findings typical of GBS and thus are considered a localized form of GBS limited to cranial nerves. Wakerley et al. analyzed 15 historical cases of PNC attributed to GBS and proposed that PNC can only be diagnosed in patients with general features of GBS who present with ocular and pharyngeal weakness in the absence of limb weakness or ataxia, with facial palsy being present or not (39). The authors further argued that it was an oculopharyngeal type of GBS that lay at the borderland between GBS and MFS (4). ABPp syndrome and PNC had something in common in terms of diagnostic criteria; some patients with ABPp syndrome in our study, for example, ABP plus ophthalmoplegia, can also be diagnosed as PNC. However, these two diseases are not interchangeable because ophthalmoplegia is not necessary for diagnosis of ABPp syndrome, and ataxia is allowed in ABPp syndrome but excluded from PNC; thus, although both are somewhere in between GBS and MFS, the coverage of ABPp syndrome is wider than that of PNC.

The current study has important implications in clinical practice of GBS. ABP could result in serious complications including aspiration pneumonia and airway obstruction. The observations in this study highlight the importance of including GBS as one of the differential diagnosis for ABP and facilitate early diagnosis of ABPp syndrome and initiation of appropriate therapy. Furthermore, the review article will also be helpful for future prospective studies aiming to determine the incidence of ABPp syndrome and its pathogenesis. Finally, the concept of ABPp syndrome has the potential to complement the current diagnostic framework of GBS: the patients with ABP who lie between GBS and MFS, but do not fulfill the criteria for the discrete subtypes of GBS or MFS and could not be explained by overlap mechanism, could be classified into the category of ABPp syndrome.

Our study is also liable to some limitations. First, as we only searched publications in English, language bias was anticipated; therefore, the incidence of ABPp syndrome may have been underestimated. Second, as a retrospective study in which most cases were from the published literature, we inevitably encountered difficulty in obtaining the details of patients, especially data of CSF testing and serial electrophysiological recordings, and thus, multicenter prospective studies should be done to further explore the characteristics of this disease. Third, the conclusion that ABPp syndrome is more reasonable to be explained by an entity of GBS than overlap mechanism is drawn from a clinical point of view, so future efforts should focus on the pathological and imaging features to obtain an overall picture of this syndrome in GBS spectrum.

## Conclusion

As the largest case series of ABPp syndrome to date, this study strengthens our understanding of this disease. With ABP being the most prominent symptom, ABPp syndrome is often accompanied by ophthalmoplegia, facial palsy, and ataxia. Subclinical neuropathy beyond cranial nerves is also found in the disease. The presence of antiganglioside antibodies against GT1a and GQ1b predicts and contributes to ABPp syndrome. From phenotypic perspective, ABPp syndrome falls between GBS and MFS and is more prone to be a separate subtype of GBS than overlap of distinct subtypes.

## Data Availability Statement

The datasets presented in this study can be found in online repositories. The names of the repository/repositories and accession number(s) can be found in the article/supplementary material.

## Ethics Statement

The studies involving human participants were reviewed and approved by the ethics committee of the Renmin Hospital of Wuhan University. Written informed consent to participate in this study was provided by the participants' legal guardian/next of kin. The patient provided written informed consent for the publication of this case report.

## Author Contributions

QC: study design and paper drafting. HC, XF, and JY: data collection and analysis. ZX: table editing and paper revision. ZL: study supervision and paper revision. All the authors read and approved the final manuscript.

## Conflict of Interest

The authors declare that the research was conducted in the absence of any commercial or financial relationships that could be construed as a potential conflict of interest.
